# Oral microbiome contributions to metabolic syndrome pathogenesis

**DOI:** 10.3389/fmicb.2025.1630828

**Published:** 2025-08-08

**Authors:** Zijun Yue, Yue Fan, Guangliang Shan, Xingming Chen

**Affiliations:** ^1^Department of Otolaryngology-Head and Neck Surgery, Peking Union Medical College Hospital, Peking Union Medical College and Chinese Academy of Medical Sciences, Beijing, China; ^2^Department of Epidemiology and Statistics, School of Basic Medicine Peking, Union Medical College, Institute of Basic Medical Sciences Chinese Academy of Medical Sciences, Beijing, China; ^3^State Key Laboratory of Common Mechanism Research for Major Diseases, Beijing, China; ^4^School of Population Medicine and Public Health, Chinese Academy of Medical Sciences and Peking Union Medical College, Beijing, China

**Keywords:** microbiology, oral microbiome, metabolic syndrome, oral pathogen, microorganisms

## Abstract

Comprising over 700 bacterial species, the oral microbiome is the second most diverse microbial community in the human body after the gut microbiome. Currently, existing review literature suggests that gut microbiome events may play a significant role in the pathogenesis of metabolic syndrome, but the role of the oral microbiome in this disease has not yet been reviewed. The oral-gut microbiome axis refers to a bidirectional regulatory system that facilitates interaction between the oral cavity and the gut through microbial pathways. The microbiota from these two sites can migrate between each other via pathways such as swallowing and blood circulation, which may participate in disease development. In addition to the oral-gut axis, the oral microbiome itself may also influence disease pathogenesis. This review examines the potential contributions of the oral microbiome in the pathogenesis of metabolic syndrome, emphasizing its impact on insulin resistance, systemic inflammation and adipokine secretion. We explore therapeutic strategies targeting the oral microbiome which hold promise as future treatments for metabolic syndrome. Future research is needed to further elucidate the causal relationship between the oral microbiome and metabolic syndrome and to develop personalized microbiome-based therapies.

## 1 Introduction

Insulin resistance, atherogenic dyslipidemia, central obesity, and hypertension are among the metabolic dysregulations that make up the metabolic syndrome (MetS; Fahed et al., 2022). It is a group of risk factors that frequently lead to increased metabolic abnormalities such as cardiovascular diseases (CVDs) and type 2 diabetic mellitus (T2DM) if left untreated (Dabke et al., 2019). MetS has caused a significant disease burden, with one-third of adult Americans suffering from MetS (Saklayen, 2018).

These days, numerous research have discovered a link between the elements of MetS and the oral microbiome, and the pathogenic mechanisms through which oral microbiome may contribute to the disease have been partially explored (Schamarek et al., 2023; Wang et al., 2023; Sanz et al., 2020; Chen et al., 2023; Mikami et al., 2021). However, a small number of studies did not find significant associations or causal relationships between the two (Kim et al., 2021; Yan et al., 2024). Nevertheless, despite these conflicting findings, a comprehensive review of the relationship between the oral microbiome and MetS remains necessary.

**Figure 1:**
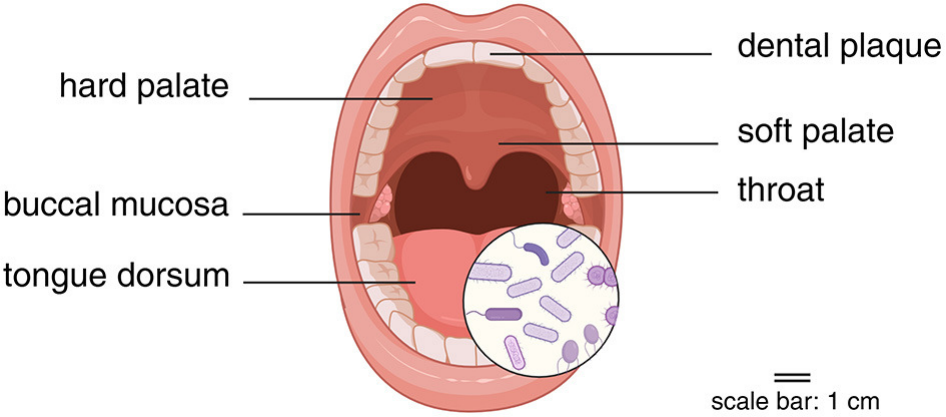
Sampling sites for oral microbiome. Created in BioRender. Yue, Z. (2025) https://BioRender.com/ngzmkfw.

Comprising bacteria, microeukaryotes, viruses, and archaea, the human oral microbiome harbors a wide variety of over 700 bacterial species, making it the second most prevalent microbial community in the human body after the gut microbiome (Costa et al., 2024). The oral microbiome is possessed of better accessibility (Baker et al., 2024) and diversified ecological niches (Lu et al., 2022), including hard palate, soft palate, dental plaque, buccal mucosa, tongue dorsum, throat, and etc. (Figure 1). It may be more suitable for large-scale and repeated sampling to study the dynamic changes of the microbiome. Interventions related to the oral microbiome (Brookes et al., 2023; Bescos et al., 2020), such as oral hygiene practices (e.g., brushing, mouthwash), may be simpler and easier to implement.

The concept of oral-gut microbiome axis refers to the bidirectional interaction between oral and gut microbiome, encompassing microbial translocation, metabolite exchange, immune signaling, and systemic health impacts (Kitamoto et al., 2020b). Oral microbes can migrate to the gut through swallowing, hematogenous spread, or mucosal transfer, thereby altering gut microbiome composition and function, which subsequently influences host metabolism, immune responses, and disease development (Kitamoto et al., 2020a; Kunath et al., 2024).

Dysbiosis of the gut microbiome has already been found to be a key factor in the pathophysiology of MetS (Dabke et al., 2019). However, no literature has yet reviewed the role of the oral microbiome in the pathogenesis of MetS.

This review attempts to clarify the possible role of the oral microbiome in the pathogenesis of MetS from a mechanistic perspective. This may provide insights for future applications of oral microbiome biomarkers in MetS risk screening. We focus mainly on oral microbiome's influence on insulin resistance, chronic inflammation, and adipokine metabolism. To note, the concept of “central inflammation” mainly refers to the hematopoietic stem and progenitor cells (HSPCs) in the bone marrow serving as a central hub to regulate the host's adaptive response to inflammation (Chavakis et al., 2019). Finally, we give a brief overview of the oral microbiome-targeted management of MetS, offering perspectives and suggestions for further investigation.

## 2 The role of oral microbiome in the pathogenesis of MetS

There are numerous intricate processes involved in the pathophysiology of MetS that have not yet been completely understood. Some studies hold the opinion that the onset of MetS occurs in the context of environmental, genetic, and dietary factors, leading to the accumulation of visceral adiposity (Fahed et al., 2022). This process results in insulin resistance, systemic inflammation, and alterations in adipokines secretion, which manifest as the phenotypes of MetS, such as obesity, dyslipidemia, and hypertension. Ultimately, these conditions contribute to an increased risk of CVDs (Fahed et al., 2022). MetS, historically termed insulin resistance syndrome, is fundamentally characterized by insulin resistance—a central phenotype that will be extensively discussed throughout this manuscript. Growing evidence demonstrates that insulin resistance interacts intricately with other key components in the pathogenesis of MetS (Lemieux and Després, 2020). Given the intersection of these mechanisms and phenotypes, this review highlights a key common molecule—free fatty acids (FFAs). FFAs are not only linked to insulin resistance, forming a vicious cycle, regulated by inflammatory factors, but also associated with adipose tissue metabolism. Therefore, the role of FFAs is mentioned in many parts of this review. This is not redundant but rather reflects the interconnected nature of MetS as a cluster of mutually influencing and interrelated conditions.

In addition to local oral diseases, it is becoming more well-acknowledged that the oral microbiome plays a significant part in the emergence of numerous systemic illnesses. Specifically, a number of extra-oral diseases, such as Alzheimer's disease, colorectal cancers, inflammatory bowel disease (IBD), rheumatoid arthritis, diabetes, obesity, and CVDs, have been linked to periodontal disease and its associated pathogens, such as *Porphyromonas gingivalis (P. gingivalis), Aggregatibacter actinomycetemcomitans (A. actinomycetemcomitans)*, and *Fusobacterium nucleatum (F. nucleatum)*. Some of these conditions are metabolic events linked to MetS (Kapila, 2021; Pirih et al., 2021; Jungbauer et al., 2022; Huang et al., 2023; Tonelli et al., 2023; Shaalan et al., 2022).

Direct pathogenic effects from local colonization of oral bacteria and a variety of indirect disease-promoting effects mediated by oral dysbiosis are the two primary, and sometimes complementary, ways by which the oral microbiome affects the pathophysiology of distal disorders (Baker et al., 2024; Imai et al., 2021).

### 2.1 The oral microbiome promotes insulin resistance through multiple mechanisms

**Figure 2:**
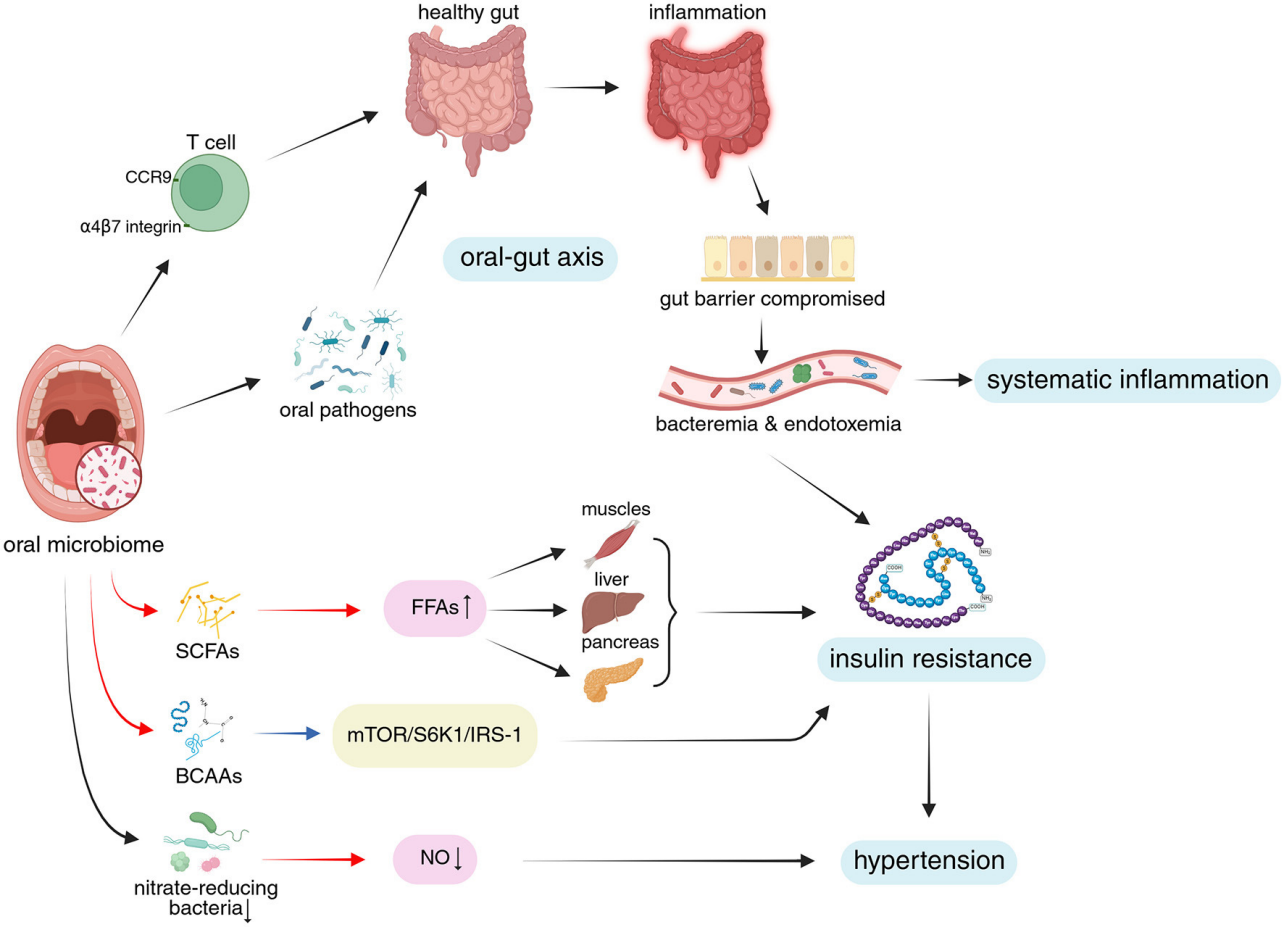
The oral microbiome influences systemic inflammation, insulin resistance, and hypertension through the oral-gut axis via various microbial metabolites and specific bacterial taxa. **Upper portion of the figure**: The illustration depicts how oral microbiome-derived immune cells and oral pathogens affect the gut via the oral-gut axis, shifting it from a healthy state to a dysbiotic, pro-inflammatory condition. This process disrupts gut barrier integrity, mediating systemic inflammation. **Lower portion of the figure**: The diagram highlights the role of oral microbial metabolites and specialized nitrate-reducing bacteria in modulating insulin resistance and hypertension through key signaling pathways, FFAs, and NO levels. Additionally, crosstalk between systemic inflammation and insulin resistance is illustrated. **Visual guide**: Black arrows: Represent progressive relationships; Red arrows: Indicate changes in metabolites; Blue arrows: Show pathway modifications; Blue background: Highlights MetS mechanisms and phenotypes discussed in this study; Pink background: Indicates microbiome-related metabolites; Yellow background: Marks relevant signaling pathways. Abbreviations: FFAs, free fatty acids; CCR9, CC-chemokine receptor 9; SCFAs, short-chain fatty acids; BCAAs, branched-chain amino acids; mTOR, mammalian target of rapamycin; S6K1, ribosomal protein S6 kinase1; IRS-1, insulin receptor substrate 1; NO, nitric oxide. Created in BioRender. Yue, Z. (2025) https://BioRender.com/0e0vmmc.

Insulin resistance represents not merely a characteristic phenotypic manifestation of MetS, but rather one of its core pathogenic mechanisms. Specifically, in the context of visceral adiposity expansion, a vicious cycle forms between insulin resistance and increased plasma FFAs. Excess FFAs act on insulin downstream signaling pathways in muscle tissue, liver, and pancreas, leading to sustained high plasma glucose levels and hyperinsulinemia, thereby inducing insulin resistance (Figure 2). In turn, insulin resistance exacerbates the condition by impairing insulin's antilipolytic effects, resulting in increased plasma FFAs (Sun et al., 2021; Yao et al., 2022; Muniyappa, 2024). This vicious cycle contributes to systemic inflammation, dyslipidemia, obesity, and hypertension, with bidirectional interactions amplifying these effects (Fahed et al., 2022).

#### 2.1.1 Oral microbiome is associated with insulin resistance

One well-known instance of oral microbiome dysbiosis is periodontal disease. Patients with severe periodontitis had significantly higher homeostasis model assessment of insulin resistance (HOMA-IR) readings than the control group in a cross-sectional study of 74 participants in India. A strong correlation between periodontitis and insulin resistance is also suggested by the greater prevalence of prediabetes, incident diabetes, and insulin resistance in the group with severe periodontitis (George et al., 2021). Other cross-sectional studies have reached similar conclusions (Pulido-Moran et al., 2017; Kalhan et al., 2025). Furthermore, compared to standard care, local periodontal therapy has been shown to lower hemoglobin A1c (HbA1c) levels in prediabetes and T2DM patients (Greggianin et al., 2023). These findings collectively suggest a precise link between oral microbiome dysbiosis and insulin resistance.

#### 2.1.2 Oral pathogens may be associated with insulin resistance through their metabolites

Through metabolic processes, the oral microbiome generates and modifies a range of fatty acids (FAs), such as short-chain fatty acids (SCFAs). These metabolites have been proven to influence the local oral environment (e.g., pH and microbial balance; Magrin et al., 2020). In an animal study, Wu et al. (2018) found that inoculation with *P. gingivalis* increased total FFAs levels in the tongue tissue and plasma of mice and altered their plasma FFAs profiles. These changes may be linked to the upregulation of *de novo* FAs synthesis pathways in the tongue tissue by *P. gingivalis*. However, there lacks definitive human studies proving that these metabolites could enter the systemic circulation and regulate FFAs levels (Figure 2). It is believed that elevated levels of plasma FFAs have a role in the development of insulin resistance, consistent with observations in human studies showing increased FFAs in T2DM patients (Weijers, 2022). Given the existing evidence, we can only suspect that metabolites from oral microbiome may indirectly exacerbate insulin resistance by influencing FFAs metabolism, potentially promoting the development of MetS. Further studies are required to determine whether and how oral microbiome-derived metabolites influence blood FFAs levels, which will help elucidate the role of oral microbial lipid metabolism in systemic diseases.

#### 2.1.3 Insulin resistance caused by high-fat diet (HFD) is enhanced by the oral microbiome

One popular model for MetS is the HFD mouse. Oral gavage of three human periodontal pathogens [*Prevotella intermedia* (*P. intermedia*), *F. nucleatum*, and *P. gingivalis*] not only caused local inflammatory bone loss in mice given HFD, but it also seemed to exacerbate insulin resistance and glucose intolerance brought on by the diet. The ability to generate particular antibodies against lipopolysaccharide (LPS) from *P. gingivalis* is linked to the underlying mechanism (Blasco-Baque et al., 2017; Figure 2). The dietary intervention used in this study may mediate insulin resistance through accumulation of visceral fat, which increase plasma FFAs levels, consistent with the central idea mentioned above, that MetS begins with the expansion of visceral adiposity (Fahed et al., 2022).

Lu et al. (2023) reported similar findings, demonstrating that mice fed a HFD for 15 weeks and inoculated with *P. gingivalis* exhibited enhanced HFD-induced insulin secretion and insulin resistance compared to those on a low-fat diet (LFD; key parameters including insulin levels and HOMA-IR). However, in this study, the levels of FFAs in HFD-fed mice did not significantly increase compared to LFD-fed mice, suggesting that the mechanisms underlying insulin resistance in MetS are not limited to the vicious cycle driven by elevated plasma FFAs (Fahed et al., 2022). Insulin resistance may also occur as a result of additional mechanisms.

Through the synthesis of branched-chain amino acids (BCAAs), the oral microbiome may worsen insulin resistance brought on by HFD. The wild-type organism, but not a BCAAs aminotransferase-deficient mutant, caused elevated serum levels of BCAAs (like leucine, isoleucine, and valine), fasting insulin levels, and HOMA-IR in a model of *P. gingivalis*-induced periodontitis in HFD-fed mice when compared to uninfected HFD-fed mice (Tian et al., 2020). This supports the idea that high levels of BCAAs in the blood could raise the risk of T2DM (De Bandt et al., 2022) by activating mammalian target of rapamycin (mTOR) and then ribosomal protein S6 kinase1 (S6K1), which phosphorylates insulin receptor substrate 1 (IRS-1) and promotes insulin resistance (Yoon, 2016; White et al., 2021; Figure 2).

In summary, the oral microbiome may enhance HFD-induced insulin resistance by generating specific antibodies against oral pathogens and producing unique amino acids, which provides new research directions for understanding the development of MetS. However, translating conclusions from HFD mouse models to human MetS research faces certain obstacles. This heterogeneity and limitations primarily stem from genetic backgrounds, dietary design, intervention time scales, and microbiome differences, among other factors (Liu et al., 2021). Approaches to bridge this gap include humanization, multi-factor induced mouse models (e.g., high-fat + high-sugar + stress), extending intervention durations, and validation using more complex animal models and human cohorts (Codazzi et al., 2024; Lone et al., 2023).

#### 2.1.4 Oral microbiome may contribute to insulin resistance through oral-gut axis

Existing research has suggested that the gut microbiome plays a part in the pathophysiology of MetS (Dabke et al., 2019), which has expanded to the oral-gut axis and its involvement in a number of disorders (Kunath et al., 2024). Under normal conditions, the oral-gut axis maintains homeostasis, preventing most oral-derived pathogenic microorganisms from bypassing barriers such as gastric acid, digestive enzymes, and intestinal epithelial tight junctions, thereby avoiding their ectopic colonization in the gut (Kunath et al., 2024). However, in certain scenarios, such as host susceptibility to colitis or pre-existing intestinal inflammation, oral pathogen enrichment and ectopic colonization in the gut may contribute to systemic diseases and insulin resistance (Yamazaki and Kamada, 2024).

Niu et al. (2024) found that the oral-gut translocation of viable *P. gingivalis* functions as a fuel connecting periodontitis and insulin resistance, involving the reduction of aryl hydrocarbon receptor (AhR) ligands and the inactivation of AhR signaling, in a periodontitis mouse model using oral ligature plus *P. gingivalis* inoculation. Insulin resistance associated with periodontitis was reduced by supplementing with an AhR agonist—Ficz (6-formylindolo (3,2-b) carbazole), where the restoration of gut barrier function may have been a significant factor. Similar to this, oral infection of HFD-fed mice with the periodontal pathogen *A. actinomycetemcomitans* resulted in changes in the gut microbiome and mediated insulin resistance, liver steatosis, and glucose intolerance in the setting of maladaptation of the oral–gut–liver axis (Komazaki et al., 2017). These evidences suggest that under adverse conditions such as host susceptibility, oral microbiome may facilitate the ectopic colonization of oral pathogens in the gut by compromising the oral-gut barrier, which may contribute to insulin resistance.

Together, the possible approaches by which insulin resistance can be modulated by the oral microbiome involve its lipid metabolism, production of oral pathogen-specific antibodies, special amino acid synthesis, and ectopic colonization through oral-gut axis (Figure 2). Together, these processes add proof for the link between insulin resistance and dysbiosis of the oral microbiome.

### 2.2 Systemic inflammation and oral microbiome dysbiosis correlates

Systemic inflammation is another critical component in the pathogenesis of MetS. Firstly, expanded visceral adipose tissue releases higher levels of pro-inflammatory factors [e.g., tumor necrosis factor-α (TNF-α), interleukin-6 (IL-6), and C-reactive protein (CRP)] while reducing anti-inflammatory factors (e.g., adiponectin). These inflammatory cytokines, in turn, promote lipolysis in adipose tissue, generating more FFAs and exacerbating insulin resistance and dyslipidemia. Additionally, inflammatory factors can directly induce insulin resistance through various downstream signaling pathways, which further sustains systemic inflammation (Fahed et al., 2022). These mechanisms collectively form the foundation of MetS pathogenesis.

#### 2.2.1 Oral microbial dysbiosis is usually accompanied by elevated systemic inflammatory markers

The oral microbiome and systemic inflammation have already been linked in a number of observational studies. In contrast to healthy controls, patients with severe periodontitis have been found to have higher blood levels of neutrophils and pro-inflammatory mediators (such as IL-1, IL-6, CRP, and fibrinogen; Schenkein et al., 2020; Teles et al., 2024). Another cross-sectional study involving 70 participants found that serum high-sensitivity CRP and fibrinogen levels were correlated with the severity of periodontitis, with the association strengthening as the severity of periodontal disease increases (Andreu et al., 2021).

Moreover, local periodontal treatment has been found to attenuate systemic inflammatory markers (e.g., CRP, TNF-α) and improve comorbid disease activity (D'Aiuto et al., 2018). In a multicenter longitudinal study involving 153 participants, researchers found that serum IL-6 levels were successfully lowered by periodontal therapy in systemically healthy patients with periodontal disease (Matsuda et al., 2024).

These findings suggest that higher levels of systemic inflammatory markers are associated with a disturbance of oral microbial balance, which may contribute to systemic inflammation.

#### 2.2.2 Oral microbiome dysbiosis can mediate systemic inflammation through various mechanisms

A wide range of studies hold the opinion that there exists a bidirectional relationship between systemic inflammation and oral microbial dysbiosis. Some indicated that systemic inflammation promote the growth of certain inflammophilic bacteria by providing tissue degradation products (Hajishengallis and Chavakis, 2021), thereby modulating oral microbial dysbiosis. However, their reciprocal correlation requires further elucidation. Here, we focus on the oral microbiome and examine evidence indicating that oral dysbiosis may induce inflammatory responses. This perspective may help establish connections between the oral microbiome and MetS from a pathogenic standpoint.

It is commonly known that oral dysbiosis and local inflammation are related. Payne et al. discovered that the dysbiotic oral microbiome caused by *P. gingivalis* could be vertically transmitted from parents to their children and stably transferred to germ-free mice. This resulted in the development of periodontitis, indicating that the dysbiotic microbiome may be a direct source of local inflammatory pathology (Payne et al., 2019; Li et al., 2023b).

**Figure 3:**
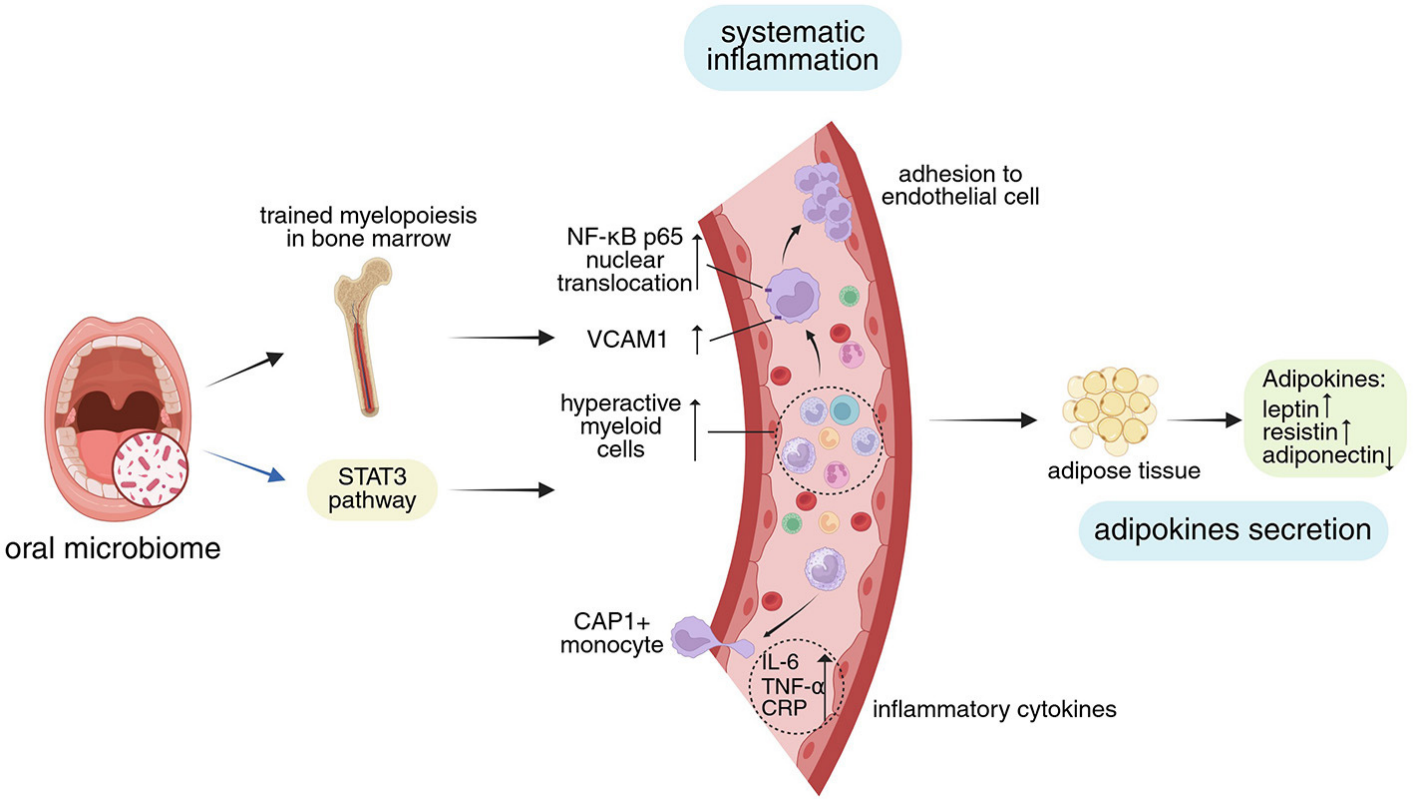
Impact of oral microbiome on systemic inflammation and adipokines secretion. The oral microbiome modulates the “central inflammation” hub function of bone marrow by altering its immune status, and influences systemic inflammation through cellular signaling pathways. Concurrently, inflammation itself regulates adipose tissue and affects adipokines secretion. **Visual guide**: Black arrows: Represent progressive relationships; Blue arrows: Show pathway modifications; Blue background: Highlights MetS mechanisms and phenotypes discussed in this study; Yellow background: Marks relevant signaling pathways; Green background: Shows adipokines secretion. Abbreviations: STAT3, signal transducers and activators of transcription 3; NF-κB nuclear factor-κB; VCAM 1, vascular cell adhesion molecule 1; CAP 1, caspase recruitment domain-containing protein 1; IL-6, interleukin-6; TNF-α, tumor necrosis factor-α; CRP, C-reactive protein. Created in BioRender. Yue, Z. (2025) https://BioRender.com/3m6ifm3.

Oral pathogens may enter the bloodstream through tissue damage caused by local inflammation, leading to transient but frequent bacteremia. This bacteremia allows oral pathogens to disseminate systemically, either directly colonizing distant sites to mediate disease or inducing endotoxemia, which stimulates the immune system to produce inflammatory cytokines and unique immune system alterations, subsequently affecting systemic inflammation (Hajishengallis and Chavakis, 2021; Plachokova et al., 2021; Figure 3).

##### 2.2.2.1 Oral microbiome may promote systemic inflammation by activating immune cells and altering signaling pathways

Hu et al. (2021) found that, in ligature-induced periodontitis (LIP) rats, peripheral blood and the brain showed apparent elevations in inflammatory cytokines (IL-1β, IL-6, IL-8, and IL-21), which are mediated by the signal transducers and activators of transcription 3 (STAT3) signaling pathway. According to a different study by Lorena et al., LIP in rats caused endothelial dysfunction and vascular inflammation, which were accompanied by elevated neutrophil counts and elevated blood levels of IL-6 and CRP (Brito et al., 2013). Periodontitis-induced activation of circulating monocytes, their increased adherence to aortic endothelial cells via nuclear factor-κB (NF-κB) p65, and the elevation of vascular cell adhesion molecule 1 (VCAM1) in the latter are considered to be contributing factors to vascular inflammation (Miyajima et al., 2014; Shen et al., 2023; Kim et al., 2023). According to these investigations on animals, the oral microbiome may encourage systemic inflammation through its specific immune responses and associated downstream signaling pathway alterations (Figure 3).

##### 2.2.2.2 Oral microbiome modulates systemic inflammation via bone marrow

Periodontal disease appears to shift the bone marrow toward a state of trained myelopoiesis, which refers to elevated levels of hyper-reactive myeloid cells with higher capacity to produce pro-inflammatory cytokines, formimg a mechanistic basis of systematic inflammation (Li et al., 2022). The correlation between periodontal and cardiovascular inflammation may be partially explained by increased hematopoietic activity, as demonstrated by ^18^F-fluorodeoxyglucose positron emission tomography–computed tomography (^18^F-FDG-PET-CT) investigations (Ishai et al., 2019; Arefnia et al., 2024), indicating that periodontitis may trigger inflammatory adaptation of HSPCs and trained immunity in the bone marrow. It can be speculated that its ability to influence cardiovascular inflammation may partially explain the phenomenon where oral microbial dysbiosis is associated with an increased risk of CVDs (Tonelli et al., 2023). In accordance with the idea of “central inflammation,” this increased bone marrow inflammatory activity might contribute to the oral microbiome's encouragement of systemic inflammation (Chavakis et al., 2019; Figure 3).

##### 2.2.2.3 The oral microbiome may affect systemic inflammation through oral-gut axis

Recent studies indicate that the oral-gut axis plays an important role in systemic inflammation associated with oral dysbiosis. Oral bacteria may disseminate via the oropharyngeal or oro-digestive route (Hajishengallis et al., 2023). As previously stated, ectopic colonization of the gut by oral organisms may mediate inflammation, alter the local microbial composition, and impair the function of the gut barrier, all of which may induce endotoxemia and modulate systemic inflammation (Kitamoto et al., 2020b; Yu et al., 2025).

From the perspective of immune cells, periodontitis, and colitis are linked via T cell priming in the periodontal tissue and subsequent T cell trafficking to the gut in mice (Kitamoto et al., 2020b). In particular, oral pathobiont-specific T lymphocytes, which proliferate during LIP, move from the cervical lymph nodes that drain the oral cavity to the gut and express the gut-homing markers α4β7 integrin (the receptor for the gut-specific vascular addressin MAdCAM1) and CC-chemokine receptor 9 (CCR9; Kitamoto et al., 2020b). These oral pathobiont-specific T cells, in which T helper (Th) 17 cells are enriched, multiply, and worsen colitis once they reach the gut (Kitamoto et al., 2020b). This represents the mechanisms linking oral microbiome to IBD. Therefore, it is plausible to believe that the oral-gut axis is essential to the procedure by which the oral microbiome mediates systemic inflammation (Figure 2).

In summary, the oral microbiome can mediate systemic inflammation by modulating signaling pathways, activating immune cells, enhancing bone marrow inflammatory activity, and altering gut microbiome homeostasis. Compared to animal studies with strictly controlled variables, human research involves certain confounding factors that require discussion. Smoking has been demonstrated to elevate systemic inflammation levels (Elisia et al., 2020), and most of the cited human studies controlled for smoking as a covariate. Even after adjusting for smoking, the studies still support that alterations in the oral microbiome itself are associated with systemic inflammation. Unfortunately, existing research has rarely thoroughly investigated baseline conditions such as dietary habits and oral hygiene, which influence the oral microbiome. It is well-established that good oral hygiene practices can reduce the incidence of dental caries and periodontitis (Zini et al., 2021; Mutluay and Mutluay, 2022). Furthermore, Liu et al. (2023) found that poor oral hygiene increases the risk of nasopharyngeal carcinoma by altering the oral microbiome. Given these overlooked potential confounders, it is reasonable to suspect that these unaccounted variables could bias the research outcomes. Future studies should aim to improve in these aspects.

### 2.3 The oral microbiome's function in the secretion of adipokines

The adipose tissue is recognized to be not only a thermoregulator and lipid storage facility, but also an endocrine organ which plays a crucial role in the pathogenesis of MetS (Fahed et al., 2022). The former primarily refers to the elevated levels of FFAs released by it, which contributes to pro-atherogenic dyslipidemia during the expansion of visceral adiposity in MetS. The latter involves various adipokines released by adipose tissue including peptides [such as resistin and plasminogen activator inhibitor (PAI)-1], hormones (e.g., leptin and adiponectin), and inflammatory cytokines (including chemerin, TNF-α, IL-6, and visfatin; Taylor, 2021; Kim et al., 2022).

**Leptin**, a multi-target organ protein hormone secreted by adipose tissue. A pro-inflammatory immune response is known to be facilitated by leptin via various immune cells (Liu and Li, 2025). The idea of “leptin resistance,” in which tissues have reduced sensitivity to leptin, was born out of the inability of high leptin levels to address the metabolic imbalance observed in obesity (Obradovic et al., 2021). Elevated levels of leptin are associated with increased risk of MetS (Pappas-Gogos et al., 2022). In an observational cross-sectional study involving 102 participants, patients with early rheumatoid arthritis exhibited higher serum leptin levels, and the presence of gingivitis or periodontal disease markers was found to influence leptin levels (Rodríguez et al., 2021). Another 6-year follow-up study in youth showed that LPS was associated with higher blood leptin levels (Perng et al., 2022).

Based on the previously discussed impact of the oral microbiome on systemic inflammation, we can speculate that oral microbial dysbiosis may contribute to abnormal leptin levels/leptin resistance through modulating systemic inflammatory factors, endotoxemia, and immune cells.

**Resistin**, a cytokine released by immune cells and adipocytes, is mostly correlated with metabolic disorders, inflammation, and insulin resistance (Kamil et al., 2023). In a single-cell RNA analysis study involving 27 participants, researchers found that the resistin pathway was intensified in individuals with periodontitis and those with both periodontitis and diabetes. By attaching to receptors and triggering several intracellular signaling pathways [NF-κB, mitogen-activated protein kinase (MAPK), and AMP-activated protein kinase (AMPK)], especially through caspase recruitment domain-containing protein 1 (CAP1)+ classical monocytes, resistin may be responsible for this phenomenon, which makes periodontitis a precursor to T2DM (Lee et al., 2023).

Conversely, the hormone **adiponectin**, which is released by adipocytes, has anti-inflammatory, insulin-sensitive, and metabolism-regulating qualities (Nesic et al., 2022; Petersen et al., 2024). Tang et al. (2022) hold the opinion that specific single nucleotide polymorphisms in the adiponectin gene may be identified as susceptibility genes for T2DM. In a cohort study involving 71 subjects, Alkan and Guzeldemir-Akcakanat (2021) found that by modifying the amounts of adipokines (IL-1β, TNF-α, leptin, resistin, and adiponectin) in the serum, saliva, and gingival crevicular fluid of obese female patients, different severity of periodontal disease affected general health. Furthermore, *in vitro* experiments observed that administration of adiponectin receptor agonists reduced the levels of TNF and IL-6 (Shinohara et al., 2022). Thus, oral dysbiosis may reduce the adiponectin levels through systematic inflammation, forming a bidirectional relationship between the latter two.

Additionally, whether periodontal therapy can reverse abnormal adipokine levels in patients with oral dysbiosis requires further validation. Multiple systematic reviews have investigated changes in adipokine and inflammatory marker levels (in serum/gingival crevicular fluid/saliva) of periodontitis patients before and after non-surgical periodontal therapy (NSPT). While some concluded that NSPT could improve dysregulated adipokine profiles (Zhang et al., 2023; Tajik et al., 2025; de Menezes et al., 2024), negative findings have been reported (Zhu et al., 2017). These discrepancies may stem from variations in inclusion/exclusion criteria across different systematic reviews, necessitating further exploration and verification through independent clinical trials.

In conclusion, the oral microbiome may influence adipokine levels through inflammatory responses, signaling pathways and the immune system (Figure 3). The changes in these adipokines seem to be synergistic—pro-inflammatory factors decrease while anti-inflammatory factors increase (as shown in Table 1), which may contribute to the pathogenesis of MetS.

**Table 1:** The role and changes of adipokines in normal condition versus oral dysbiosis.

**Adipokines**	**Normal condition**	**Oral dysbiosis**
Leptin	Pro-inflammatory	↑ and/or leptin resistance
Resistin	Pro-inflammatory	↑
Adiponectin	Anti-inflammatory	↓
Visfatin	Pro-inflammatory	↑
Chemerin	Pro-inflammatory	↑
Omentin-1	Pro-inflammatory	↑

### 2.4 Oral microbiome may mediate hypertension through modifying nitric oxide (NO) metabolism

NO is essential to the onset and advancement of hypertension. As a key vasoactive molecule, NO controls endothelial function, inflammatory responses, and vascular tone, all of which affect blood pressure (Bryan, 2022).

According to a study using data from the National Health and Nutrition Examination Survey (NHANES), there may be a connection between the prevalence of hypertension, periodontal disease, and oral health (Li et al., 2023a). In a similar vein, a short-term prospective cohort study of 2,588 Japanese students also discovered a strong correlation between hypertension and periodontal disease (Kawabata et al., 2016).

The positive effects of nitrate-rich meals on blood pressure may be mediated by oral bacteria, especially nitrate-reducing bacteria (e.g., *Streptococcus* and *Veillonella* genera). These bacteria play a part in NO production, and their reduction caused by oral dysbiosis may impair NO-mediated vasodilation, which, at least in part, leads to the development of hypertension (Alzahrani et al., 2021; Barbadoro et al., 2021). Meanwhile, dietary nitrate supplementation can alter the oral microbiome in individuals with hypertension, exerting potential prebiotic effects (du Toit et al., 2024). Furthermore, longitudinal studies suggest that excessive oral hygiene practices (such as prolonged use of over-the-counter antimicrobial mouthwashes) may reduce the abundance of oral nitrate-reducing bacteria, leading to decreased NO production and potentially increasing hypertension risk (Joshipura et al., 2020). This finding was confirmed in exploratory experiments, where 7-day use of chlorhexidine mouthwash altered the oral microbiome of healthy subjects, reduced nitrite concentrations in both saliva and plasma, and resulted in elevated systolic blood pressure (Bescos et al., 2020).

Meanwhile, the development of hypertension is also partially influenced by insulin resistance. Studies have shown that during insulin resistance, the vasodilatory effects of insulin are reduced, while its activation of the sympathetic nervous system and renin-induced sodium reabsorption remains intact. These mechanisms collectively contribute to the pathophysiology by which insulin resistance promotes hypertension (Fahed et al., 2022). Therefore, based on the earlier discussion of how the oral microbiome may promote insulin resistance, it is reasonable to suspect that the oral microbiome may indirectly affect the development of hypertension by inducing insulin resistance.

Moreover, the impact of periodontal therapy on blood pressure has been explored in numerous randomized controlled trials (RCTs) and systematic reviews (Zhou et al., 2017; Shang et al., 2025). Evidence of varying quality suggests that periodontal treatment may have short-term beneficial effects on subjects' blood pressure, but there is a lack of long-term evaluation of this intervention (Muñoz Aguilera et al., 2020; Sharma et al., 2021; Meng et al., 2024; Orlandi et al., 2022). The systematic review by Luo et al. (2021) concluded that current evidence is insufficient to demonstrate definitive blood pressure-lowering effects from periodontal therapy, highlighting the need for longer-term, high-quality studies to validate the benefits of oral health improvement on hypertension.

In conclusion, it is reasonable to suspect that the oral microbiome plays a potential role in the development of hypertension by producing NO and indirectly through insulin resistance (Figure 2).

## 3 Targeting oral microbiome for the treatment of MetS

Current research on microbiota-based therapies primarily focuses on restoring gut microbiome homeostasis, mainly through the supplementation of probiotics, prebiotics, fecal microbiota transplantation (FMT), and localized microbial modulator. Exploration of oral microbiome-based therapies follows a similar approach, though current research in this area remains limited.

Research has shown that eating habits have a big impact on the oral microbiome's composition (Vach et al., 2022). A high-fiber diet aids in weight loss for obese individuals, improves blood glucose levels and insulin sensitivity in both non-diabetic and diabetic individuals, and reduces blood pressure and serum cholesterol levels, offering benefits for various components of MetS (Anderson et al., 2009; Lepping et al., 2022). By lowering the amount of *Alloprevotella* in the oral microbiome, Sato et al. (2024) found that a high-fiber diet with a high 12-component modified Japanese diet index (mJDI12) may enhance overall health. However, there are certain challenges in modulating the oral microbiome through dietary interventions. Individual genetic backgrounds, lifestyle variations, and compliance issues may hinder both the adoption of dietary changes and their effectiveness. Moreover, current research lacks long-term dynamic studies on oral microbiome changes following dietary modifications, and the mechanisms by which specific nutrients regulate key microbial species remain unclear (Kerstens et al., 2024). Additionally, the metabolic transformation of dietary components under the influence of the oral microbiome has not been fully elucidated (Bai et al., 2025; Shoer et al., 2023). Future research should focus on elucidating the tripartite interaction mechanisms among the oral microbiome, diet, and host, while developing tailored dietary modification strategies for different eating patterns and investigating their long-term effects.

Strategies using probiotics/prebiotics to restore oral microbiome homeostasis have been explored. Probiotics that produce nisin or nisin itself can change the oral microbiome for the better, reducing periodontal destruction and host immune responses (Gao et al., 2022; Nguyen et al., 2020). By dramatically reducing the messenger RNA (mRNA) expression of pro-inflammatory cytokines (IL-1β, IL-6, and TNF-α) in the brain that are raised by periodontal infection, these probiotics also help neuroinflammation that resembles Alzheimer's disease and is brought on by periodontal disease (Zhao et al., 2023). According to Rosier et al., people with diabetes and hypertension may benefit from taking supplements containing nitrate/symbiotic combos (nitrate + nitrate-reducing probiotics). However, individual variations in oral microbiome's nitrate-reduction capacity exist. Some people may therefore benefit directly from nitrate as a prebiotic because of their microbiota's innate capacity to significantly reduce nitrate, while others may benefit more from symbiotic pairings (Rosier et al., 2020). Therefore, it is necessary to emphasize personalized microbiome-based therapies in the future. Two double-blind, placebo-controlled trials from the same research team (Yarahmadi et al., 2024; Bazyar et al., 2020) collectively demonstrate that combining symbiotic supplementation with NSPT may help improve inflammation, antioxidant status, periodontal health, glycemic control, and reduce LDL- cholesterol in patients with T2DM and chronic periodontitis. The study by Zorina et al. (2017) likewise revealed similar results. Therefore, from the perspectives of inflammation and microbial metabolism, supplementing with probiotics/prebiotics helps restore oral microbiome homeostasis and promote beneficial metabolic processes, potentially offering benefits for MetS. Nevertheless, potential risks associated with probiotic/prebiotic supplementation warrant careful consideration, including unintended microbiome alterations that may lead to opportunistic infections, or horizontal transfer of antibiotic resistance genes (Ji et al., 2023; Merenstein et al., 2023). Future long-term longitudinal studies are imperative to thoroughly evaluate the safety profile and intervention robustness of this approach (Wieërs et al., 2019).

Corresponding to FMT, the oral microbiota transplantation (OMT) has been introduced (Nascimento, 2017; Beikler et al., 2021). In animal experiments, Xiao et al. (2021) showed that OMT helps mice with head and neck cancer who had radiation-induced oral mucositis. Subsequent clinical research by Goloshchapov et al. (2024) found that maternal saliva transplantation prevented severe chemotherapy-induced oral mucositis, accompanied by changes in the composition of the patient's oral microbiome, with no negative transplant-related events noted. However, the implementation of OMT currently faces significant challenges. Unlike the well-established FMT, there is no internationally recognized standardized protocol for OMT (Bokoliya et al., 2021). Critical gaps exist in multiple aspects, including donor screening, transplantation routes, administration protocols, complication prevention, colonization dynamics of transplanted microbes, and ethical oversight (Allegretti et al., 2020). These obstacles primarily stem from the insufficient depth, breadth, and long-term scope of oral microbiome research itself. It is anticipated that they can be gradually resolved as the field advances in the future. Despite these limitations, OMT remains a promising candidate for future oral microbiome-based interventions.

These encouraging findings suggest that microbiota-based therapies targeting the oral microbiome are a promising and worthwhile direction for exploring MetS treatment. However, further research is needed to refine aspects such as dosage, treatment duration, efficacy, adverse effects, and long-term outcomes.

## 4 Discussion

In this review, we have summarized the possible contributions of the oral microbiome to the pathogenesis of MetS and described therapeutic explorations targeting oral homeostasis. Nonetheless, there is no denying that oral microbiome dysbiosis and MetS are correlated in both directions (Dame-Teixeira et al., 2025). Future research should prioritize well-designed RCTs to establish causal relationships between the oral microbiome and incident MetS. Additionally, integrating multi-omics approaches (e.g., metagenomics, metabolomics, and proteomics) could further elucidate the underlying mechanisms and strengthen the evidence for causality.

Longitudinal lifespan studies in both animal models and human populations are required to forecast the long-term consequences of microbiome-based interventions (Hsu et al., 2021; Depommier et al., 2019). Optimization of more comprehensive and robust animal models of MetS are needed as well.

Moreover, the structure of oral microbiome varies across different niches (Baker et al., 2024). Most of the current research on oral microbiome comes from studies on periodontal disease and dental plaque, while other specimens need to include saliva, oral rinses, buccal mucosal, dorsum tongue swabs, and etc. (Jiao et al., 2022; Arweiler et al., 2021; Chan et al., 2021) The link between the oral microbiome at distinct oral niches and different diseases should be one of the main focuses of future research.

To sum up, this study methodically describes how the oral microbiome may contribute to the pathogenesis of MetS, offering important new information for future studies on the connection between systemic disorders and the oral microbiome. Though further research is required to convert these findings into therapeutic applications, studies on the oral microbiome show promise for conquering MetS.
